# Retail Stores Policies for Marketing of Lobsters in Sardinia (Italy) as Influenced by Different Practices Related to Animal Welfare and Product Quality

**DOI:** 10.3390/foods7070103

**Published:** 2018-07-02

**Authors:** Giuseppe Esposito, Daniele Nucera, Domenico Meloni

**Affiliations:** 1 Department of Veterinary Medicine, University of Sassari, Via Vienna 2, 07100 Sassari, Italy; gsesposito@uniss.it; 2 Department of Agriculture, Forest and Food Science, University of Turin, Via Verdi 8, 10124 Turin, Italy; daniele.nucera@unito.it

**Keywords:** crustaceans, supermarkets, aquarium, ice

## Abstract

The aim of the present study was to evaluate the marketing policies of lobsters as influenced by different practices related to product quality in seven supermarkets located in Italy. Retailers were divided in two categories: large scale and medium scale. The two groups were compared to screen for differences and to assess differences in score distribution attributed to different practices related to product quality. Our results showed no statistical differences (*p* > 0.05) between the two categories. Lobsters were often marketed alive on ice and/or stocked for long periods in supermarket aquariums, highlighting the need to improve the specific European regulations on health, welfare, and quality at the market stage. Retail shop managers should be encouraged to develop better practices and policies in terms of marketing of lobsters. This will help in keeping the animals in good health and improve product quality at the marketing stages.

## 1. Introduction

In the last decades, the global total consumption of seafood products has increased: this has resulted in a rapidly growing demand for these products, especially in emerging markets [1]. A recent FAO (Food and Agriculture Organization) report has shown that in 2014, the global total production of fish, crustaceans, mollusks, and other aquatic animals reached 158 million tons [2]. Lobsters, spiny lobsters, marine and freshwater prawns, and crabs, whose breeding practices have shown continued growth over time, especially for their high nutritional and commercial importance [3,4]. The world quantity of crustaceans caught in marine fisheries has grown from 5377.062 in 2006 to about 5751.000 tons in 2014 [2]. Nowadays according to FAO statistics, there is a growing worldwide market demand for the crustaceans and this increase was also recorded for lobster species that has grown from about 253,000 tons in 2006 to about 293,800 tons in 2014 [2]. In Europe and Italy, lobsters are available throughout the year and are subjected to permanently increasing prices in the market [5]. *Homarus americanus* is caught especially in North America and it is one of the major economic resources of these coastal communities [4]. The total landings of this species have substantially increased in the last decades [5,6], including 159,814 tons in 2014 [2]. The annual catches of *Homarus gammarus* (the most valuable and preferred commercially species, fetching very high prices) instead was 5194 tons in 2014. The latter species is mainly captured in the North-eastern Atlantic countries (United Kingdom, Ireland, Channel Islands, and France), while, in the Mediterranean Sea, the catches decreased [6]. This resulted in an increase in imports of American lobster, estimated at about 4387 tons annually introduced in Italy [4,7]. Consumer perceptions on the respect of health, welfare, and quality of lobsters at the market stages are steadily increasing. The question of consumer-oriented quality policies is a key challenge in a competitive market: the objectives of food business operators and large-scale retailers should correspond to consumers’ demands [8]. Despite the millions of lobsters placed on the EU market per year, at present, in the current legislation, there are not specific regulations. The marketing stage is a crucial step for the respect of the welfare of these crustaceans and impact the organoleptic quality of their meat. In some countries (e.g., Norway, Australia, and New Zealand), the decapods crustaceans are afforded specific guidelines or regulations on animal welfare [9,10,11]. The overall framework for European Union (EU) action on animal welfare is set out in the EU Animal Welfare Strategy 2012–2015 [12]. Thus, animal welfare has become a primary objective of Community Policies because stress factors and poor welfare can lead to increased susceptibility to diseases among animals [13]. According to EU Regulation n. 853/2004 [14], this can pose risks to consumers—for example, through common food-borne infections. The welfare of food-producing animals depends largely on how they are managed by humans [12]. Different factors can impact on their welfare (e.g., housing, space and crowding, transport conditions, and slaughter methods) [13]. Nevertheless, in Directive 2010/63/EU of the European Parliament [15], invertebrates (including the lobsters) are excluded. Recently, the conservation and marketing modalities of the lobsters have been subjected to legal interpretation, and the scientific community showed a growing ethical concern toward their animal welfare. Many scientific studies showed that these animals show a complex behavior and physiological responses to noxious stimuli that provide evidence of their capacity to experience pain and stress [16,17,18]. Thus, handling and conservation of these animals during marketing are practices that must meet minimum welfare requirements [19,20]. Furthermore, according to EU Regulation n. 853/2004 [14], the crustaceans sold alive are defined as “food of animal origin-fishery products” and, during marketing, must be kept at a temperature and under conditions that do not jeopardize food safety and quality. Keeping these animals alive and promoting their welfare decreases the microbiological risk and promotes a better nutritional organoleptic quality. Some species of crustaceans, including lobsters, are traditionally marketed alive and stored for long periods in supermarkets or restaurants’ aquariums. The current legislation does not fix limits on the parameters of storage, prohibits feeding (otherwise storage would become breeding), and ensures the safety of operators with the claws clasped. In restaurants and supermarkets, third parties usually manage the aquariums carrying out inspections and maintenance twice a month or every six months. Some water parameters (temperature, pH, and nitrites) are managed by the retail staff [21]. The aim of the present study was to evaluate the marketing policies of 2 lobster species (*H. americanus* and *H. gammarus*) as influenced by different practices related to animal welfare and product quality in seven large- and medium-scale supermarkets linked to international and national supply chains and located in Sardinia (Italy).

## 2. Materials and Methods

### 2.1. Supermarkets Description and Sampling Plan

From December 2015 to June 2016, a fact-finding survey was carried out at the fish shops of seven supermarkets (SA to SG) linked to international and national supply chains and located in North-western Sardinia (Italy). The majority of the supermarkets included in this study (80%) were international chain supermarkets (a supermarket that shares both its name and operations with other supermarkets worldwide); the remaining 20% were Italian chain supermarkets. Retailers were divided in two categories: large scale *n* = 4, SA to SD) and medium scale (*n* = 3, SE to SG). Seven (70%) of 10 eligible supermarkets contacted agreed to participate. Eligible large-scale supermarkets were recruited via a preliminary visit. Managers of the fish shops were told that the data and pictures would be collected anonymously. In order to evaluate the conservation and marketing modalities of *H. americanus* and *H. gammarus*, three unannounced sampling days per month were scheduled in every supermarket for a total of 21 sampling visits (147 sampling visits on the whole).

### 2.2. Sample Collection

During every sampling day at each large-scale retail shop, visual observations of employees’ work routine and selling procedures were conducted discretely. An evaluation form was drawn up reporting the supermarket characteristics, practices, and policies in terms of conservation and marketing of the European and American lobsters (placed dead or alive on the ice or stored in aquariums). Additional information regarded the most sold species of lobsters, the simultaneous presence of different species of crustaceans in the aquarium and the presence of separated spaces, the number of lobsters per aquarium, the dimension and capacity of the aquarium, the presence of sea bottom replacements (sand, rocks, stone chippings), the presence and the type of water filter, and the maintenance and management of the aquariums and the water cleanliness. The main observational data have been evaluated qualitatively as present/absent (yes or not). To assess differences in adequacy of aquariums’ dimensions and water cleanliness, a score has been attributed to the dimension of the aquarium (1, small, 1050 × 550 × 910 + 550 mm; 2, big, 1550 × 650 × 930 + 550 mm) and water cleanliness (1 poor; 2 mediocre; 3 good; 4 excellent). Altogether, 100 pictures were taken with a digital camera Nikon D3100 (Nikon Corp., Tokyo, Japan). The evolution of some water parameters, such as temperature and pH, has been verified by the examination of the aquarium’ management sheets compiled by the retail staff. Data were averaged.

### 2.3. Statistical Analysis

The two categories of retail shop, large-scale (*n* = 4) and medium-scale (*n* = 3), were compared in order to screen for differences in frequency of positive answers to questions related to practices and policies in terms of conservation and marketing of lobsters. Moreover, differences in score distribution attributed to adequacy of aquariums’ dimensions and water cleanliness have been assessed. The first analyses on qualitative variables was performed by means of chi-square tests. In particular, for the analyses of 2 × 2 tables the Yate’s corrected formula was applied using EPI info 6^®^ software (CDC-Centers for Diseases Control and Prevention, Atlanta, GA, USA). The score variables were investigated by means of Mann-Whitney U test performed by SPSS vs. 12.0^®^ for Windows (IBM SPSS Statistics, Armonk, New York, NY, USA). Results were considered significant when *p* < 0.05 for all the tests performed.

## 3. Results and Discussion

Data were collected in seven large- and medium-scale supermarkets showing a comparable and rather approximate management of the lobsters with difficulty identifying the various batches. The results showed no statistical differences in terms of conservation and marketing modalities between the two categories of retail shop (*p* > 0.05). This compromises the internal traceability system, and very often, these animals experienced prolonged stay, fast, and stress. Table 1 presents observational data and scores on large and medium-scale retail stores policies and practices for marketing of American Lobster and European Lobster.

### 3.1. Large- and Medium-Scale Supermarkets’ Policies and Practices for Marketing

The fact-finding survey found that the most sold species of crustaceans are lobster (*H. gammarus* and *H. americanus*), spiny lobster (*Palinurus elephas*), and crab (*Cancer pagurus* and *Maja squinado*). The best-selling of these crustaceans were the lobster species. Our results showed different types of conservation and marketing modalities of lobsters (Table 1) that are not in compliance with animal welfare. Lobsters placed alive on the ice and/or stored in aquariums had claws ligatured with clasp to avoid competitiveness, cannibalism, and claw damage due to their aggression and to guarantee the safety of operators. This practice determines muscle atrophy and also causes interference with normal animal ethology [19]. Despite the fact that all the large- and medium-scale retail stores included in our study were provided with specific aquariums, at the market stage, the lobsters were anyway placed alive on ice (Table 1). During the period of peak demand (from December to January), the amount of lobsters placed alive on ice was 67% in retail store F. Conversely, the lower percentage (17%) was registered in retail store D. Placing the lobsters directly on ice is an unsuitable storage method since the direct contact with the ice determines strong stress conditions (e.g., jump in temperature, anaerobic stress, hypoxia etc.) so much that it prevent their vitality [19]. In some fish shops (C and D), we found that the lobsters were mostly placed on the market inside overcrowded aquariums. No statistical differences in terms of aquariums’ dimensions were pointed out between the two categories of retail shop (*p* > 0.09). This condition could produce a progressive worsening of water quality and contextually increases the microbiological risk while it also favors a poor organoleptic and nutritional quality of the products. More than 50% of the retail shops included in our study showed low scores (poor or mediocre) in terms of water cleanliness. No statistical differences were pointed out between large- and medium-scale retail shops (*p* > 0.09). In some retail stores (A and F), other species of fish and/or crustaceans were found in the same aquarium of lobsters. The confinement for long periods with other individuals [22] is one of the many stressor factors that can cause the mobilization of energy substrates [23] as the release of lactate in the hemolymph and hyperglycemia [24]. Furthermore, the exposure to a direct and intense light as happens in many fish shops may cause a stress condition that reduces the survival rates [19]. These animals manly nocturnal, and during the day, they take refuge in holes or crevices that are located within a range of water depths of 35–40 m with a low light intensity [25]. None of the retail stores included in our study followed a single guideline for the management of lobsters in the aquariums. Since it was not possible to apply the all-in all-out system, it was not possible to differentiate the newly introduced lobsters to those already present in the aquarium. According to previous studies [21], the presence of any dead lobster inside the aquarium was detected daily and immediately removed.

### 3.2. Temperature and pH of Water in the Aquariums

In all the supermarkets, the measurement of water parameters (temperature and pH) for the aquariums was carried out by retail staff on a weekly or daily basis and reported on dedicated management sheets. The temperature and pH measurements (Table 2) were in accordance with specific ranges indicated by aquarium suppliers. All the temperature and pH parameters were relatively constant over time. The correct management of the water parameters appears to be an important factor in maintaining vital lobsters in the aquariums and promoting their welfare [21].

## 4. Conclusions

Animal welfare is a longstanding concern of the European Union (EU). In the last few years, it revised the general rules concerning the protection of animals, irrespective of species. Recently, the scientific community has shown a growing ethical concern toward the category of invertebrates as part of animal welfare. The current legislation does not have ethical guidelines or regulations that set limits on the parameters of the storage and marketing of live lobsters [20]. These crustaceans can experience pain, and it is considered ethically unacceptable to continue subjecting these animals to inhumane treatments [17]. For these reasons, the handling of lobsters at the marketing stage must be conducted humanely [9]. On the contrary, the storage of lobsters in aquariums at low temperatures can continue for extended times without being fed [5,21]. Keeping lobsters at low temperatures reduces their metabolism, so that they are not forced to use their energy reserves [21]. Otherwise, the suffering of the lobsters inside the aquariums is greatly accentuated: the nervous system is 100% operational and sensitive. Previous studies showed that lobsters suffer protein degradation of both thick and thin filaments due to the stress induced by fasting [21]. In most supermarkets, lobsters are stored in overcrowded water tanks and experience prolonged stay, fast, and stress with chemical and microbiological pollution of water and loss of organoleptic and nutritional quality. Food business operators should pay greater attention to both physical and chemical parameters of the water and try to shorten the staying time of the lobsters in the aquariums. Rational cooling, purging to reduce nitrogenous waste products, and banding of claws with clasp are highly recommended at the marketing stages [3]. However, the prolonged ligature of the claws causes muscle atrophy, inhibition of nutrition (if natural), and interference with the threat/defense behavior. In addition, the application of the band in molding animals can distort and weaken the claws [19]. The claws clasp should be a useful tool to enhance the internal traceability system to identify lobsters of the same batch using a single color for the elastic placed around the claws or using the batch matrix printed on the elastic [21]. Rapid environmental changes may cause stressful conditions for crustaceans, increasing the vulnerability to bacteria, and reducing the immune response [5,26]. The use of ice as a cooling agent during the marketing should be carefully evaluated: the direct contact between ice and alive lobsters might prevent their vitality. A rational use of ice needs a basal layer of ice and a grid shelf with live lobsters resting above. Out of the water, there is a constant dulling of the sensorium, and the lobsters are in a pre-agonal state. The main limitation of this study is that the assessment of large- and medium-scale retail stores for the marketing of lobsters as influenced by different practices related to animal welfare and product quality was done only by the observation method. However, this is one of the audit/inspection methods according to ISO 19011/2011 guidelines for auditing management systems [27]. In order to guarantee ethically acceptable crustaceans conditions, retail shop managers should be encouraged to develop better practices and policies in terms of marketing lobsters. Specific regulations on quality, health, and welfare of these crustaceans at the market stage are needed. This will help in improving lobster quality and survival and, at the same time, the performance of the large- and medium-scale retail stores.

## Figures and Tables

**Table 1:** Differences in conservation and marketing modalities of lobsters between large- and medium-scale retail stores in Sardinia (Italy).

Features	Large-Scale Retail Stores				Medium-Scale Retail Stores			Statistical Significance
	A	B	C	D	E	F	G	
Lobsters placed on ice alive	No	Yes	No	No	Yes	Yes	No	Not significant (*p* > 0.4)
Dead lobsters placed on ice	Yes	Yes	Yes	No	Yes	Yes	No	Not significant (*p* > 0.9)
Lobsters stored in aquariums	Yes	Yes	Yes	Yes	Yes	Yes	Yes	Not significant (*p* > 0.9)
Simultaneous presence of different species of crustaceans	Yes	No	No	No	Yes	No	No	Not significant (*p* > 0.9)
Number of lobsters per aquarium	10–15	5–10	3–5	3–5	5–10	5–10	5–10	#
Dimension of the aquarium *	1	2	1	2	2	1	2	Not significant (*p* > 0.5)
Capacity of the aquarium (lt)	300	535	300	535	535	300	535	#
Presence of water filter and aerator	Yes	Yes	Yes	Yes	Yes	Yes	Yes	#
Water Cleanliness **	4	4	2	1	1	2	4	Not significant (*p* > 0.5)
Single tank	Yes	Yes	No	No	Yes	No	Yes	Not significant (*p* > 0.9)
Separated spaces for different species	No	No	Yes	Yes	No	Yes	No	Not significant (*p* > 0.9)
Presence of sea bottom replacements	No	No	No	No	Yes	No	No	Not significant (*p* > 0.4)

* 1, small, 1050 × 550 × 910 + 550 mm; 2, big, 1550 × 650 × 930 + 550 mm; ** 1 poor; 2 mediocre; 3 good; 4 excellent. # = statistical significance has not been evaluated.

**Table 2:** Mean values of temperature and pH in the aquariums of large- and medium-scale retail stores in Sardinia (Italy).

Water Parameters	Large-Scale Retail Stores				Medium-Scale Retail Stores		
	SA	SB	SC	SD	SE	SF	SG
pH	7.5	7.7	7.6	7.5	7.8	7.8	7.5
Temperature	8 °C	8.5 °C	7.5 °C	7.5 °C	7.5 °C	8.5 °C	8 °C

SA, SB, SC, SD = Large-Scale Retail Stores; SE, SF, SG = Medium-Scale Retail Stores.
